# Will New York Lead?

**Published:** 1898-05

**Authors:** 


### WILL NEW YORK LEAD?

The time is opportune, the situation is ripe for a strong, united, and aggressive movement for recognition with rank of our army veterinarians. Some of those who have given many hours of thoughtful work to this movement in the past are now resident in the Empire State. The National Guard of the State has some of our best veterinarians in its ranks. Higher education has well fitted the graduates of the colleges of this State for these positions, some of which are now held by her sons; and would it not be fitting for the veterinary profession, the colleges, the associations, and every associated interest to enter at once upon the work necessary to accomplish this great end. *Will New York lead?*

